# Rage against the mean: a perspective on measuring fitness of individual phage particles

**DOI:** 10.1038/s44298-026-00187-4

**Published:** 2026-04-04

**Authors:** Jyot D. Antani, Paul E. Turner

**Affiliations:** 1 Department of Ecology and Evolutionary Biology, Yale University, New Haven, CT USA; 2 Center for Phage Biology & Therapy, Yale University, New Haven, CT USA; 3 Quantitative Biology Institute, Yale University, New Haven, CT USA; 4 Program in Microbiology, Yale School of Medicine, New Haven, CT USA

**Keywords:** Biological techniques, Computational biology and bioinformatics, Microbiology

## Abstract

Traditional bacteriophage methods measure population averages rather than individual particle variation. This perspective advocates for approaches to quantify trait variation in phage particles. Emerging techniques in optical microscopy and flow cytometry can reveal previously-masked phenotypic heterogeneity, offering unprecedented insights across phage infection cycle: binding kinetics, genome entry, replication, coinfection dynamics, and particle stability. This shift from population averages to individual variation represents a critical frontier for phage biology and biotechnology.

## Introduction

Virology is a relatively young science, established in 1898 when Martinus Beijerinck discovered that tobacco mosaic virus was the causative agent of a plant disease^1^. The largely submicroscopic sizes of viruses made them easy to miss and essentially ‘invisible’ to biologists, until microscopy and imaging technologies advanced to make their visualization more routine. Despite these achievements, a key portion of the virus world remains difficult to measure and therefore mainly invisible: variation in traits among individual particles in a virus population.

Like all biological populations, individual viruses (particles) can vary in their traits, and the expectation is that the variant(s) with fitness (performance) characteristics relatively favored by natural selection will evolve to dominate the virus population. This outcome is well known in viral diseases of humans such as influenza and COVID, where new viral variants can evolve because they are better capable of overcoming host immune defenses, antiviral medicines, and vaccines^2,3^. However, such strains are recognized after they become very common (rise to high genotype frequencies), and long after they first appear as mutants in a virus population. Virology is still challenged to develop tools and approaches that measure differences in traits among individual particles in a virus population. Fundamentally, our knowledge of population biology and evolution is advanced by understanding the phenotypic (trait) differences among variants and how processes such as natural selection and genetic drift cause changes in genotype frequencies over time, measured in generations. Thus, despite a deep appreciation for the power of viruses to evolve^4–6^, we lack efficient ways to measure differing attributes – particularly the trait(s) that contribute to fitness – of individual particles within a virus population^7^.

Viral diseases understandably capture public health attention when dangerous variants spur epidemics and pandemics, bringing problematic strains into focus when they become very common. However, only a tiny fraction of viruses on Earth are important in human disease. The vast majority are bacteriophages (or phages, for short) – viruses that infect and replicate only within bacterial cells, first hinted by Frederick Twort in 1915 and best described by Félix d’Hérelle in 1917^8,9^. Phages seem to constitute the most abundant and diverse biological entities on our planet, with an estimated 10^31^ phage particles globally^10^. Phages seem to outnumber even bacteria (estimated as 10^30^ cells globally), and the interactions between phages and host bacteria likely occur in the vast majority of Earth’s ecosystems, ranging from deserts to oceans, deep-sea vents to mountaintops, and most other locations where life can thrive (we note that extreme environments often contain Archaea with their specific viruses—sometimes called archaeal phages, but these viruses are not reviewed here). Phages are also present in microbiomes of animals and plants, though they remain less studied than their bacterial co-inhabitants. Given this ubiquity, it seems plausible to assert that phage infections of bacterial host cells represent the most numerous biological interactions on Earth.

The teeming abundance of phage-bacteria interactions warrants greater efforts to develop high-throughput measures of trait differences among individual phage particles, to better elucidate how and why phage variants can be evolutionarily favored. Additionally, our ambitions for using phages in biotechnology offer strong motivations to develop these tools^11^. Phages are relatively easy and inexpensive to culture, especially lytic phages that tend to efficiently infect and replicate in bacteria, killing (lysing) susceptible host cells in the process^12^. This potential for lytic phage populations to grow exponentially on target host bacteria makes them attractive options for developing solutions to applied problems. For example, bacteria can cause biofouling that contaminates surfaces, including biofilms and slimes which impair machinery and clog pipes, sometimes producing metal corrosion^13,14^. Phage biotechnology may be capable of addressing such problems, if phages can be leveraged to combat biofouling^15^. Moreover, the rise of antimicrobial resistance (AMR) presents a global public health threat, as bacteria are increasingly observed to resist traditional chemical drugs, raising alarm bells that alternatives are sorely needed. Phage therapy is an old idea pioneered by d’Hérelle soon after describing phages in 1917^9^, but chemical antibiotics quickly gained popularity by the 1940’s and World War II after Alexander Fleming accidentally discovered them in 1928^16,17^. Nevertheless, the earlier practice of using phages to treat bacterial infections did not wane in countries like Russia, Poland, and the former Soviet Republic, Georgia. Given the current widespread threat of AMR bacteria, there is resurged interest in phage therapy across Western and other nations^12,18–20^. Altogether, phages are recognized as potential tools for solving human problems, making it vital to determine which variants possess traits best capable of biocontrol and other technology ambitions.

A common challenge for better elucidating biology of phages and developing these viruses in biotechnology is that many of the research methods from the mid-1900s remain popular today, practical for measuring the mean trait values in phage populations. These techniques can mask useful information on individual variation existing within phage populations, making it harder to learn about biological differences between particles and to discern which candidate phage could be ideal for problem-solving. Intraspecies trait variation often drives crucial ecological phenomena^21^. Even as phage synthetic-biology and engineering methods quickly advance^22–27^, these techniques may not incorporate measures of particle-level variation that could minimize (or possibly eliminate) occurrence of less-desirable variants in the manufacturing process. The past two decades have seen rapid development of tools for studying the genotypes and phenotypes of individual bacterial cells, revealing details of varying physiology, motility, and other traits, even among isogenic cells^28–36^. Whereas analogous techniques for phages remain challenging to develop, hampering the ability to understand trait-heterogeneity within phage populations that would benefit both basic knowledge and applied research on Earth’s most plentiful inhabitants.

**Fig. 1:**
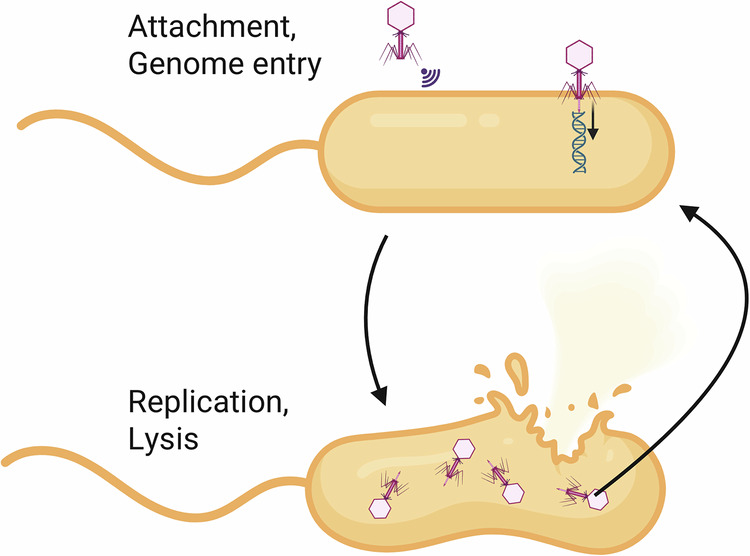
Lytic infection cycle of phages. The lytic cycle commences with phage recognition, reversible interactions, and irreversible attachment with specific bacterial surface receptors. Attachment is followed by phage genomic entry into the host cytoplasm. Subsequently, the phage commandeers host cellular resources such as proteins, genomes, and metabolic processes, to facilitate replication, transcription, and translation of new viral components. Following assembly of the progeny viral particles, the bacterium undergoes lysis, liberating newly formed phage particles to continue the infection cycle in neighboring susceptible cells. Created in BioRender. Turner, P. (2026) https://BioRender.com/okshlai.

In this perspective, we focus on the importance of developing high-throughput measures of trait variation among individual phage particles. To do so, we traverse the infection cycle of a lytic phage (Fig. 1): starting with cell-surface interaction, entry of nucleic acid into the cell, intracellular replication and progeny production via cell lysis (destruction), possible consequences of cellular co-infection, and finally particle stability outside of the cell. (Other phage replication strategies exist, including temperate viruses that are transmitted during cell division, occasionally lysing host cells; and filamentous phages that typically reproduce by secreting progeny from the cell without killing it.) We review traditional approaches for estimating phage traits, and some exciting new methods that reveal how individual phage particles can vary in traits important for relative fitness. Our goal is to provide illustrative examples, rather than to produce a comprehensive review, as exciting new research in phage biology and biotechnology continues to advance rapidly. We discuss why these increased efforts should include improved measures of trait variation among individual phage particles, to advance fundamental understanding of phage biology, ecology, and evolution, and to enhance development of phages as problem-solvers in biotechnology applications.

## Phage interactions with cell surfaces

Phage particles move passively through environments which may contain a variety of cell types, both suitable (permissive) and non-suitable for infection. Because viruses lack metabolism, a phage does not actively move; rather, particles can freely diffuse and bombard cells, experiencing either reversible binding/unbinding or irreversible attachment to structure(s) on a cell surface^37^. If receptor-binding protein(s) of a phage do not permanently attach, the phage diffuses away, perhaps encountering other cell types. Alternatively, a phage can permanently bind to a cell, potentially followed by entry or injection of the virus nucleic acid (DNA or RNA) into the cell, with possibility (but not assurance) for successful downstream steps of the infection cycle.

The attachment (adsorption) step is crucial for a phage to match with its suitable host cell. Phages target specific structures on the host-cell surface such as transmembrane channels (e.g., porins) composed of individual proteins or complexes, appendages (e.g., pili and flagella), and sugar moieties (e.g., lipopolysaccharides) or capsules which surround the cell body^12,38,39^. Beyond broad characterization as gram negative versus positive, bacteria are otherwise extremely diverse in their surface composition. If a phage adsorbs (permanently attaches) to a host that it cannot infect, this constitutes a ‘dead end’ infection for the virus particle. Albeit some phages can reversibly attach to non-host bacteria and “hitchhike” to host-rich regions, while some others undergo lysis from without (a seemingly rare phenomenon where adsorption of numerous phage particles causes cell lysis without phage progeny production); these mechanisms operate with significantly lower efficiency than direct infection^40–42^. It is crucially important that a phage finds a correct host for successful infection, since diffusion (not active motility) is the only effective means for the phage to explore its environment.

### Traditional estimates of phage adsorption

**Fig. 2:**
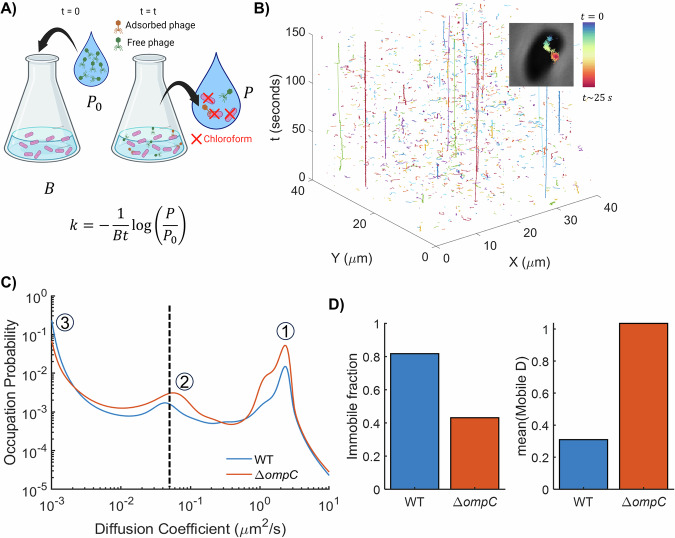
Measurements of phage adsorption. **A** In the traditional adsorption assay, ${P}_{0}$ phages are mixed with bacteria, and unattached phages ($P$) are enumerated at various time-points ($t$) afterward. An ensemble estimate of the adsorption rate $k$ or effective adsorption rate ${k}_{{eff}}={kB}$ is measured by obtaining fits of the depleting $P/{P}_{0}$ to an assumed model of phage association with bacteria, e.g., ${dP}/{dt}=-{kBP}$. **B** We developed a Microscopic Phage Adsorption Assay, where we tracked individual phages interacting with live, immobilized bacterial cells^51^. An example phage trajectory is shown as inset on top right corner, whereas the main figure indicates thousands of trajectories moving over time (the vertical dimension) into, out of, and within the microscopic field of view. **C** We used a machine-learning based particle tracking algorithm that infers a distribution of diffusion coefficients, $D$, from single-particle trajectories^53^. We applied it to trajectories of T4 phages interacting with host (WT) cells and cells lacking the cognate receptor OmpC. We observe roughly three diffusive states: an unbound/freely diffusing state ① – peak at high $D$ corresponding to phages diffusing in 3D, a bound state ③ – peak at low $D$, and an intermediate state ② – likely corresponding to diffusion on the bacterial surface while reversibly interacting with host factors. **D** From these distributions, we calculated the immobile fraction and mean diffusion coefficient of the mobile fraction (using a threshold of $D=0.05\,\mu {m}^{2}/s$), which helped us parametrize the biophysics of phage attachment. The immobile fraction is lower for the phages interacting with cells lacking the receptor, while the mean $D$ for mobile trajectories is higher, as compared to when interacting with WT cells. Parts of the figure created in BioRender. Turner, P. (2026) https://BioRender.com/eb20jdz.

The classic adsorption assay was developed in the early 20th century, in the same decade as the initial discovery of phages^43^. This assay often involves mixing in liquid a known number of phages and an excess of bacterial cells, to increase the probability that each particle attaches to a cell and to decrease the likelihood that multiple particles co-infect the same cell. The goal of the assay is to estimate ‘disappearance’ of phage particles in solution over time (Fig. 2A). As phages attach to host cells, the number of free phage particles should deplete over time, whereas this disappearance is unexpected in controls where phages are mixed with non-permissive hosts (e.g., those lacking known phage-binding proteins). Aliquots of the mixture are sampled at different time-points (e.g., every few minutes for 15 min total), and bacteria are removed via filtration or using a chemical such as chloroform. An adsorption rate constant ($k$) is calculated based on a model of adsorption, generally a first order association model of phages ($P$) and bacteria ($B$) as follows:

$$\frac{{dP}}{{dt}}=-{kBP}$$

Other proposed models are used to interpret data from classic adsorption assays, e.g., those that account for reversible attachment steps, or first-order models approximating the excess of bacterial concentration as constant and thus a part of the effective adsorption rate^44^.

This classic approach for measuring phage adsorption remains widely used by phage biologists. However, it has two major limitations: it requires substantial manual labor and supplies, and more critically, it only provides an ensemble mean estimate for the phage population. This approach cannot measure attachment of individual particles or capture any heterogeneity in the adsorption process.

### Newer methods for measuring phage adsorption

Microscopic measurements of individual phages attaching to bacterial cells may provide a single-phage readout of adsorption. Electron microscopy is traditionally and routinely used to characterize structures of phages and their components^45^. Technical advances in cryo-electron tomography have proven extremely useful in identifying the structures and roles of various phage components in adsorption^46,47^. However, electron-based microscopic approaches require sample immobilization, thus precluding visualization of dynamic processes. Optical microscopy provides an exciting opportunity to measure the dynamics of phage adsorption, where particles can be fluorescently labeled via approaches like chemical labeling or genome engineering to fuse fluorescent proteins with phage components. An early optical microscopic study visualized phages hybridized with quantum dots adsorbed to *Escherichia coli* cells, revealing that most of the seven tested phages localized at cellular poles during infection of live bacterial cells^48^. Shortly after, Rothenberg and coworkers tracked individual phage-λ particles interacting with host cells, revealing that the phages reversibly interacted with phage-receptor LamB protein units – wrapped helically around *E. coli* cell surfaces – before reaching receptor-rich cellular poles^49^. In this work, they labeled phages by fusing capsid protein gene *gpD* with the fluorescent protein EYFP^50^, leveraging the extensive research history on phage λ molecular biology. This phage-labeling approach is not yet generalizable due to genetic engineering challenges in non-model species of bacteria and phages. Advances in phage genome editing and synthetic phage genome assembly are expected to address these limitations significantly^22–27^.

Circumventing the genome engineering challenges, we recently developed a chemical-labeling-based technique to measure individual phage particle attachment to bacterial cells (Fig. 2B)^51^. We immobilized bacterial cells on glass surfaces, introduced phages labeled with a non-specific fluorescent dye, and recorded time-lapse movies of individual phages forming bright fluorescent foci. We used a fluorescent dye that labels lysine residues and amine groups exposed on the phage surface, deliberately avoiding genome-labeling dyes, which allowed visualization of particles that had injected their genomes while capsids remained attached to cells. Through computerized particle tracking, we obtained x-y trajectories of the phages. Thus, we measured the distributions of dwell time (duration of each trajectory), the time that a phage particle interacts with bacterial cells. Notably, we observed massive heterogeneity in dwell times, suggesting a much higher fraction of reversible interactions than previously assumed^44^. In addition, the distributions of dwell times featured a heavy tail resulting in a power law-like distribution instead of an exponential distribution characteristic to stochastic processes described by a single characteristic time. Rather, our observations suggest that phages may follow a continuum affinity model, characteristic of variability in the dissociation constant within a population^52^.

Probing attachment of individual viruses allows examination of biophysical quantities associated with virus-cell interactions. We applied a recent machine learning algorithm to infer diffusion coefficient distributions, fraction of immobile particles, and the mean diffusion coefficient for the mobile fraction from our single-phage trajectories^53^. We recorded interactions of coliphage T4 with wildtype bacteria and a receptor-less (Δ*ompC*) mutant strain at a high temporal resolution (100 frames/second) to improve the inference. The results from this previously unpublished experiment are shown in Fig. 2C. We also applied this algorithm to our datasets from the published work discussed above^51^—these experiments were performed at a lower temporal resolution (30 frames/second), and the results are shown in Fig S1. From the quantitative inferences, we note that bacterial mutants lacking phage receptors had a significantly lower fraction of immobile phage trajectories. Additionally, the mobile fraction of phages in these assays with Δ*ompC* mutants exhibited a higher diffusion coefficient, indicating that phages move faster on surfaces lacking receptors. Future developments in single particle analysis will enhance our ability to infer biophysical parameters of phage attachment. We also aim to improve throughput by integrating our approach with advances in microfluidics and small-volume handling technologies.

## Phage genome entry into the host cell

After a phage is irreversibly attached to its receptor on the host surface, the phage genome enters the host cell. For widely studied model coliphages such as T-phages and λ, the genome is injected into the host cell. For example, following the receptor-recognition and irreversible binding mediated by long tail fibers of T4, short tail fibers anchor the outer membrane, and a conformational change in the base plate signals contraction of the tail sheath and forcing of viral DNA into the cell^54^. On the other hand, in phage λ with a non-contractile tail, after host-recognition protein J binds to LamB, phage DNA passes through the trans-outer-membrane porin LamB and the mannose permease complex in the inner membrane^55^. Not all phages inject their genomes into the cell. For instance, RNA phage ϕ6 infects *Pseudomonas* cells by first fusing its envelope with the host cell’s outer membrane after which the nucleocapsid penetrates the inner membrane^56^.

### Traditional estimates of phage genome entry

The first measurement of genome injection dates to the famous Hershey-Chase ‘blender’ experiment, where phage DNA was labeled with radioactive phosphorous isotope (^32^P) while protein was labeled with radioactive sulfur (^35^S). An ordinary kitchen blender was used to detach phages from bacteria, followed by centrifugation to separate cell pellet from phage suspension. ^32^P was found inside the cells infected with phage while ^35^S remained in the suspension, proving that virus DNA, not protein, is the genetic material^57^. Other early studies similarly used ^32^P or ^14^C to probe phage genome entry^58–60^. Southern hybridization assays have also been used to investigate phage genome entry into the cell^61,62^. All of these studies determined key steps of genome entry of model coliphages, and informed several models of the physics of genome entry mechanisms, reviewed elsewhere^63^. Electron microscopy and tomography have depicted mechanistic details of some phages that experience genome injection, often at the scale of individual domains or amino acid residues^46,54,64^.

Albeit, the genome entry experiments described above measured the timescales of total DNA content from all phages entering all cells within the culture vessel, rather than probing entry of individual virus particle genomes. Such assays cannot measure any heterogeneity in rates, fidelity, or other parameters relating to genome injection among individual particles. While electron microscopy can visualize individual particles, measuring sub-second dynamics is challenging for both radiolabeling and electron microscopic approaches due to complex sample preparation requirements.

### Newer methods for measuring phage genome entry

**Fig. 3:**
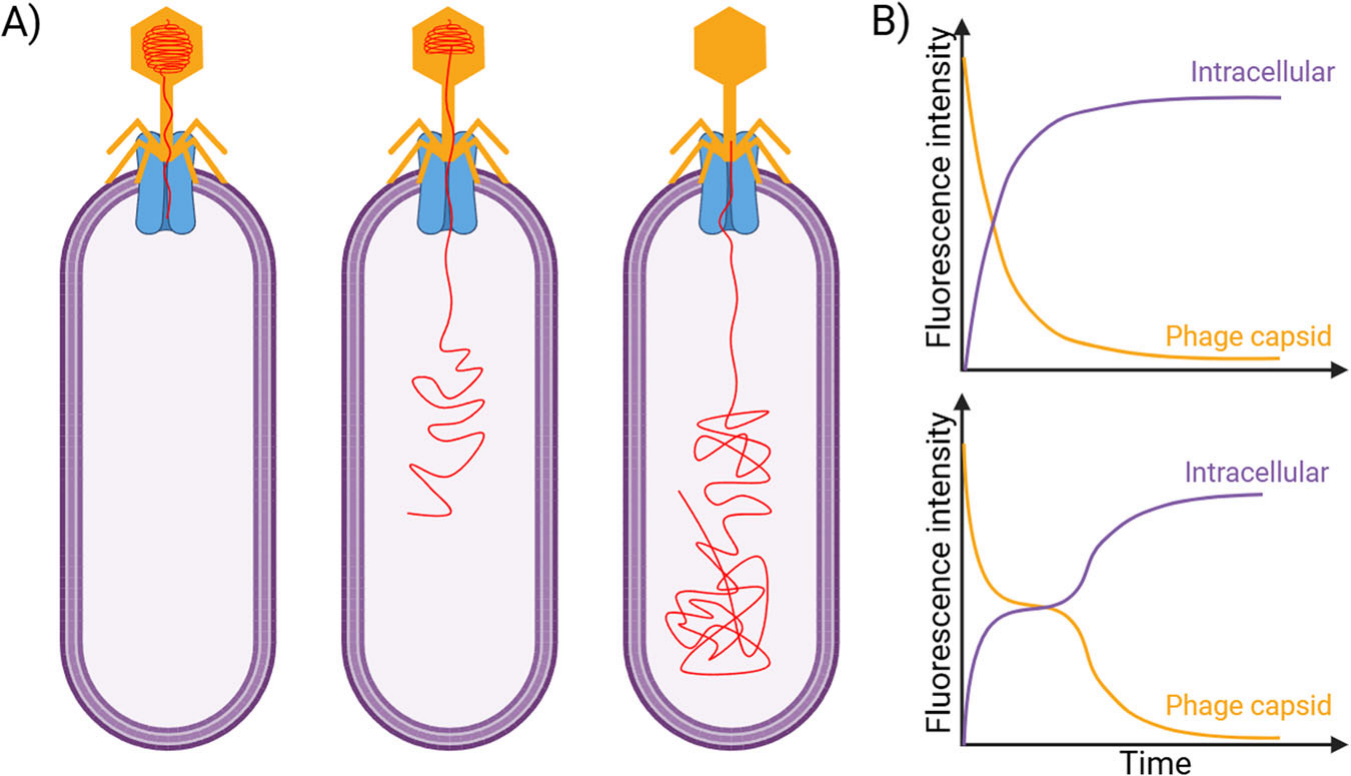
Measurements of phage genome entry into bacterial cells^65^. **A** Phage λ DNA was labeled with DNA stain SYTOX Orange, and translocation of the DNA into host cell was visualized with fluorescence microscopy. **B** Upon genome ejection from the phage, the total fluorescence signal inside the phage capsid declined, while the signal inside the cell simultaneously increased. While some genomes entered the cells rapidly [top], others stalled after translocation of a portion of the DNA [bottom] before proceeding to complete translocation. Created in BioRender. Turner, P. (2026) https://BioRender.com/4yg5xhe.

Optical microscopy-based observations of phage genome entry can provide insights into the heterogeneity in genome entry in a phage population. Van Valen and coworkers performed a microscopic equivalent of the Hershey-Chase experiment: they labeled phage λ genomes with a DNA stain (SYTOX Orange) and visualized the entry of individual phage genomes into cells^65^ (Fig. 3A). They quantified fluorescence intensity inside phage capsid and within the cell, and were able to observe that as fluorescence signal from the capsid decreased, signal inside the cell increased in tandem, serving as a readout of DNA translocation. The results revealed significant variability in genome translocation from individual phage particles: some phage genomes were 80% translocated into the cells within 1–5 min while others paused or stalled for up to 7 min after a smaller fraction of the genome was transferred (Fig. 3B). While example traces are shown in the schematic in Fig. 3B, individual traces had significant variation^65^. Such heterogeneity for individual phage genome translocations pairs would have been impossible to observe without optical microscopy experiments. These authors were also able to calculate the velocity of DNA ejection, parametrizing the existing models of phage DNA translocation^63,65^. The occurrence probabilities of stalling events and the intraspecies variation observed in this work may drive crucial events in genome entry dynamics.

## Replication and lysis (burst size)

A key component of relative fitness in any biological system is the number and quality of offspring (progeny) produced within a given time period, relative to other variants in the population. After genome entry into the cell cytoplasm, host metabolism is diverted for the energy to drive replication of phage genetic material and production of viral proteins and enzymes, utilizing ribosomes and other hijacked components of the cell’s machinery. The newly replicated viral DNA or RNA along with structural and other proteins then self-assemble into new phage particles. For lytic phages, the progeny particles may experience additional steps that precede formation of ‘mature’ virions, which can be released simultaneously from the cell in a ‘burst’: destruction (lysis) of the cell often aided by a phage-encoded lytic enzyme. The ‘burst size’ is defined as the number of infectious progeny particles that exit from a single cell when lysis goes to completion^66^. The time-duration associated with various steps of the infection cycle are other quantitative viral traits which include lysis time, latent period, eclipse period, and rise period- each defined below. While it is increasingly appreciated that phages sometimes encounter anti-phage defense systems within the bacterial cell^67,68^, and that some phages have evolved counter-defense systems^69,70^, here we focus on variation in phage traits if intracellular replication proceeds successfully. Burst sizes range from roughly ten particles to several thousand progeny produced per lysed cell^66,71,72^. Burst size can also depend on several factors such as host strain genotype and physiology, abiotic environmental conditions, such as temperature and pH^66,73^, which sets the expectation that burst size for individual phage particles within a population should be highly variable too. However, this variation is hard to measure with classic methods.

### Traditional estimates of phage reproduction

**Fig. 4:**
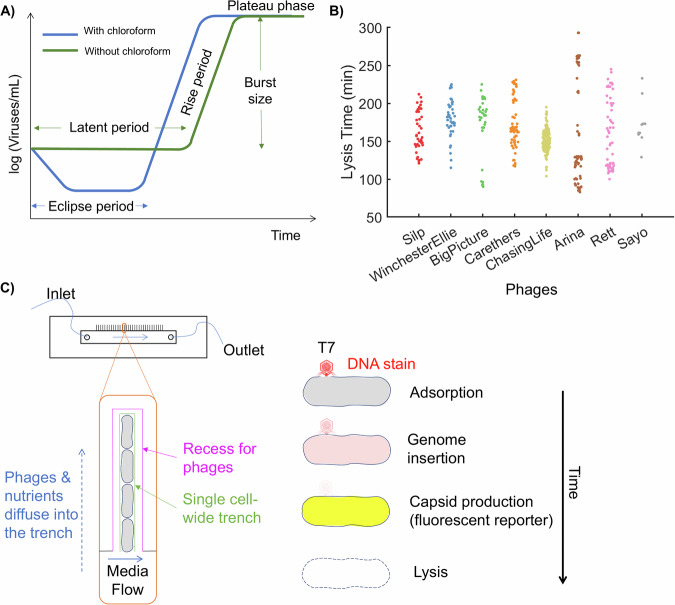
Measurements of phage replication traits. **A** In the traditional one-step growth curve, the densities of infectious viruses are estimated over time in a mixed phage-bacteria culture. The time from infection to the start of rise in viral counts provides an estimation of the *eclipse period* or *latent period*, depending on whether or not chloroform is used to lyse the cells. This period is followed by a steady logarithmic increase in viral numbers, often termed as the *rise period*. After the viral count reaches a plateau, the ratio between the final count and the initial count provides an estimation of the average *burst size*, i.e., number of viral progeny produced by each infected cell. **B** We used phase-contrast time-lapse microscopy to measure the *lysis time*, defined for single cells, as the time between infection and lysis^77^. Single cell lysis time distributions for *E. cloacae* infected with a panel of eight phages are indicated in the plot. **C** Wedd and colleagues modified a microfluidic ‘mother machine’ to measure the time-scale associated with various steps in the replication cycle of coliphage T7. *[Left]* A microfluidic ‘mother machine’ consists of a central channel where fresh media with nutrients is continuously flowed in. Single-cell-wide trenches (green) allow visualization of individual lineages started from the same mother cell. Wedd et al. added a recess (black) to allow phages to diffuse into the trenches (blue). *[Right]* A DNA stain (SYTOX Orange dye, indicated in red) and a capsid reporter (mVenus NB, a Yellow Fluorescent Protein, indicated in yellow) allowed the visualization of phage adsorption, genome injection into the cell, and the assembly of new viral particles. Phase-contrast microscopy revealed the time to lysis (indicated with dashed lines for cell boundary).

A fundamental technique used to study the replication cycle of lytic phages is the one-step growth curve (Fig. 4A). This method provides crucial information about the timing and efficiency of average phage reproduction within a bacterial host population. The technical considerations and critical steps of this experiment are discussed elsewhere^66,74^. Briefly, the process begins by mixing bacteria with phages at a low multiplicity of infection (ratio of phage particles to bacterial cells), to increase the probability that each bacterial cell is infected by only one phage. After permitting time for phage attachment, the mixture is then diluted to reduce the likelihood that progeny particles exiting the cell will bind to other cells and initiate infection. Aliquots are sampled at regular intervals and plated on agar to estimate the number of infectious phage particles. The resulting trace of infectious particles over time typically exhibits three distinct phases: the latent period, where the number of infectious phages remains constant as they replicate inside host cells; the rise period, characterized by a rapid increase in phage numbers due to cell lysis and progeny virion release; and the plateau phase, where phage numbers stabilize after all infected cells have lysed. This curve allows determination of key parameters such as latent period duration (time from starting infection to start of lysis) and burst size (average number of phages released per infected cell). When chloroform is used to lyse bacterial cells before plating the aliquots, it liberates packaged phage particles from unlysed cells. The time until a rise in such particles is observed is called the eclipse period, which is shorter than the latent period. Modern techniques like qPCR have also been employed to quantify phage genomes during the one-step growth curve, providing an estimate of phage genomes instead of infectious particle counts.

The quantification methods discussed above, including qPCR, rely on average estimates from all the phages and infected bacteria within the reaction volume. Recent modeling work has shown that variability in single-cell lysis time can heavily bias the measurements in one-step growth curves: under the assumption of Gaussian-distributed lysis times, broader distributions (with larger standard deviation) yield a shorter latent period while narrower distributions with the same mean yield a longer latent period in the one-step growth curve experiments^75^. Recently, a detailed theoretical and numerical analysis of phage traits predicted that lysis time variability is an important determinant of phage fitness^76^.

### Newer methods for measuring phage replication

Many improvements are possible for measuring phage replication cycles in individual cells. Optical microscopy can be employed to measure single-cell lysis time—the time from phage infection to lysis. We recently developed a generalizable microscopic approach to measure single cell lysis time distributions when traditional one-step growth curve measurements proved challenging^77^. We infected *Enterobacter cloacae* cells with phages and incubated them on a coverslip-sealed agarose pad under a microscope. We then used phase-contrast microscopy to capture time-lapse images of the cells over several hours, visualizing growth and lysis of individual cells. Analyzing the time-lapse movies, we recorded lysis time-points for individual cells, which yielded lysis time distributions within the same population. Using this approach, we obtained single-cell lysis time distributions for a panel of jumbo phages (phages with genomes larger than 200 kb, which are known to have long replication cycles) infecting *Enterobacter cloacae* (Fig. 4B)^77^. Tartari and coworkers recently reported a similar approach where they confined phage-infected *E. coli* within optical traps integrated with photonic crystal cavities, measuring not only lysis time but also proposing that nanofabrication technology integrated with optical microscopy may enable estimation of single-cell burst sizes^78^.

Wedd and colleagues recently reported the development of a microfluidics-based approach to measure replication-related time-scales for individual T7 viruses interacting with host *E. coli* cells^79^. They employed a microfluidic ‘mother machine’, where media is flowed through a wide channel while cells grow in a linear fashion within orthogonally arranged narrow trenches (Fig. 4C). The researchers modified the trenches to allow viruses to diffuse into the single-cell-wide trenches. They labeled T7 phage genomes with a nucleic acid intercalating stain, enabling visualization of individual virions adsorbing to cells and injecting their genetic material. Next, a fluorescent capsid production reporter was used to determine the time when individual capsids were synthesized within the cells as the virus replicated its genome. Finally, phase-contrast microscopy was used to identify the time of cell lysis. Through these combined methods, Wedd and coworkers successfully measured the distributions of timescales for different steps in the phage life cycle (Fig. 4C)^79^. While the authors successfully encoded the fluorescent capsid production reporter into the well-characterized coliphage T7, expanding this approach to non-model bacterial and phage systems remains challenging. Ongoing advances in phage genome engineering are expected to make this more feasible in the future, broadening the applicability of this technique beyond well-studied coliphages^22–27^.

Measurements of single-cell burst sizes require combining various single-cell isolation techniques with viral counting approaches. Kannoly and coworkers recently measured heterogeneity in burst sizes of induced phage-λ lysogens^80^. They diluted lysogens (bacterial cells carrying phage genome integrated within their chromosomes) such that each well in a 96-well plate contained either one cell or no cells, immediately after chemically inducing the lytic cycle. Then, they incubated the cells for the viral genome to replicate, lysed cells using chloroform at various time-points, and counted the number of virions in each well, thus measuring burst sizes for single cells. They parametrized an empirical model describing the relationship between burst size and the incubation time after chemical lysis, and reported that the noise in these two phage traits was not correlated. While effective for lysogenic phages, this dilution-based method would likely require extensive experimental optimization for lytic phages, which are of interest for many therapeutic and biocontrol applications. Dominguez-Mirazo and colleagues used flow cytometry to sort individual phage-infected cells into single wells and measured single-cell burst sizes through plaque assays^81^. They were also able to infer distributions of single-cell lysis times, and showed direct evidence that this approach can be applied to a non-model environmentally-sourced bacterium from *Synechococcus* species, infected with a lytic cyanophage^81^. Technological advances in flow cytometry are expected to enable the sensitivity to count individual viruses^82,83^. When combined with single-cell techniques, flow virometry (counting viruses in a flowing medium) offers promising opportunities for high-throughput quantification of burst sizes at a single-cell resolution.

## Co-infection and its consequences

Depending on the relative abundances of viruses and cells in a proximate neighborhood, a single cell may be infected by multiple viruses. Because viruses do not have active mobility, they usually encounter bacterial surfaces through diffusion, and the arrival times of viral particles are thought to be stochastic^84^. However, viruses may alter the physiology of the cell once they interact with the cell, such that the infecting virus reduces (or eliminates) the possible entry and infection by other virions^85^. These mechanisms such as superinfection exclusion, their ecological and evolutionary consequences, as well as the open questions in this subfield are reviewed elsewhere^85,86^. We focus on scenarios where multiple particles can enter and replicate in the same host cell.

### Traditional estimates of phage traits during co-infection

Traditionally, consequences of cellular infection by multiple phage particles have been studied by co-culturing cells with viruses at high multiplicity of infection (MOI), i.e., a high ratio of infective virus particles to cells. These studies have revealed the importance of mechanisms such as superinfection exclusion (prophage blocking cell entry by a lytic phage), as well as mutualistic ‘cooperation’ between viruses that enhance each other’s intracellular replication and defective interference (also known as viral “cheating”), where a virus reduces the productivity of other co-infecting viruses. For instance, when lineages of RNA phage ϕ6 were experimentally evolved at high MOI (under frequent co-infection) cheater variants fixed despite lowering overall fitness of the population; a result consistent with the classic ‘Prisoner’s Dilemma’ game theory solution that shows selfishness can invade a population although cooperation is more beneficial^87,88^. In this same system, a limit to co-infection prevents more than 2-3 phage ϕ6 particles from entering the same cell, perhaps because co-adsorption by multiple virus particles overwhelms *Pseudomonas syringae* cell integrity, causing the host to die prematurely before phage replication is completed^89^.These results suggest that new mechanisms similar to classic superinfection exclusion await discovery, which would impact whether multiple particles co-infect a host cell; e.g., recently it was shown that a prophage encodes a superinfection exclusion protein that inhibits the function of its receptor such that other phages cannot infect the cell harboring the prophage^90^.

### Newer methods for measuring consequences of phage co-infection

Optical microscopy and flow cytometry are increasingly employed to probe co-infection of the same cell by multiple phages. Nguyen and colleagues used fluorescence microscopy to enumerate host-adsorbed λ phages and phage genomes replicating within host cells, observing that not all adsorbed phages had their genomes enter into cells^91^. As the number of co-infecting (adsorbed) phages increased, the probability and rate of genome entry for every phage decreased. Combining optical trapping experiments with fluorescence microscopy, the authors found that the cell membrane is depolarized additively by each adsorbed phage, which in turn impedes the entry of other genomes into the cell^91^.

Our group recently used flow cytometry to identify cells infected with virions of two different phage species, and determined whether each virus was able to produce progeny following cell lysis^92^. We observed that co-infected cells often produced particles of one—but not both—of the co-infecting ‘parental’ phage species. Supplemented with other experiments, this approach helped us conclude that a bacterial population composed of only a single genotype could facilitate the coexistence of multiple different phage species, thus violating Gause’s competitive exclusion principle (assumption that two competing species cannot permanently coexist on a single limiting resource). One possible explanation is that different phages can coexist if each has a relative fitness advantage over the other, when infecting otherwise identical host cells that are in different physiological states^92^. Such phenomena are more easily revealed when studying individual bacterial cells in experiments that control whether one versus multiple phages undergo infection.

## Phage particle stability outside of host bacterial cells

‘Phage stability’ is often characterized for newly discovered phages, as phages can differ in their susceptibility to degradation by natural stressors. Common environmental conditions known to accelerate phage degradation include extreme pH and temperatures, desiccation, mutagens such as ultraviolet light, and chemical stressors^93,94^. These stressors are thought to either impair the structural proteins involved in infection (thus inhibiting attachment or genome entry) or cause mutations in the nucleic acid packaged within the capsid. Model phages which have been repeatedly passaged in the lab may be selected to robustly infect host bacteria across different culture conditions^82^. Whereas, non-model phages can show an order of magnitude or more decreased titer (infectious-particle concentration) when stored in the lab (even at 4 °C, a popular storage condition), presumably due to differences between their typical ecological niche and the lab environment. Therefore, 99% or more of newly produced phage particles can lose their infectivity even under benign conditions. For this reason, particle stability is a crucial focus in applied uses of phages in biotechnology, to maximize phage titers in manufacturing and to preserve these high densities during storage.

Phage stability within macro-organisms is relevant for better understanding of viruses within microbiomes, as well as to develop phages for therapy and prophylaxis when addressing disease concerns in humans and other animals, and in plants^95^. For organisms with innate and adaptive immune systems, a concern is that these defenses could neutralize delivered phage particles, which might differ for particular phage species and whether the host is immune-compromised. The interaction of phages with the human immune system is a particularly active area of research, and reviewed elsewhere^96^. Below we mention a less-studied phenomenon: interactions between individual phage particles and eukaryotic cells, and approaches for studying variability in these interactions.

### Traditional estimates of phage particle stability

Phage stability is usually characterized by enumerating infectious particles through traditional plaque assays on bacteria lawns. Typically, a phage population is subjected to differing levels or times of exposure to a stressful perturbation, such as various temperatures or pH conditions. The concentration of infectious phages (e.g., PFU/mL) is recorded before/after exposure—sometimes for only a few minutes if the condition is highly lethal, or for days/weeks when determining stability during storage in presumably benign conditions^93,94^. Classically, this is used to measure a ‘reaction norm’ which is a general term for estimating changes in traits (including survivability of individuals) across a range of environmental conditions^97^. Thus, the measurements are averages of survival of infectious particles from a population, rather than actual visualization of the degradation of individual particles. While such approaches are practical for determining average lethality of different environmental conditions, they mask the ability to explore variation in the responses of individual phage particles to the tested stressor. Structural biology and evolutionary genetics studies of viruses are not often combined, therefore we lack general information on how the morphology of a virus affects its fitness (often referred to as ‘form and function’ studies when these coincide). Historically, phage stability research has emphasized mainly the role of differing abiotic conditions for particle degradation. But several studies within the last decade have used modern tools to examine phages interacting with biological factors, especially eukaryotic cells and their products^98–101^. For example, various phages are observed to be engulfed by mammalian cells at different rates, with the possibility for these viruses to be broken-down into components used as resources for eukaryotic cell metabolism^99^. Thus, phages are unknown to replicate within eukaryotic cells, but they may be used as resources, and there is a concern that phage engulfment can reduce doses delivered during therapy.

### Newer methods for measuring phage stability

In general, the classic double-layer agar method has been the most useful technique for enumerating infectious particles by visualizing and counting plaques formed on confluent lawns of permissive host bacteria incubated for 24 h or more. This technique is low-throughput and often requires long incubation times for slowly growing bacteria. In order to increase the speed and throughput of infectious particle-counting, alternative approaches are emerging through developments in liquid-handling robotics and microfluidics, where automated processes increase throughput and plaque formation by phages can be detected at time points before plaques become visible to the naked eye—thus reducing the incubation time before readout^102–106^. Additionally, visualizing the dynamics of individual phage particles interacting with host cells (using the approaches mentioned in earlier sections) under different environmental conditions may reveal how the stochastic probabilities associated with various steps of infection change over time according to exposure conditions.

Barr and colleagues performed several studies to understand the interaction of individual phage particles with mammalian cells and their secreted mucus^98–101^. Early work from this group tracked single coliphage-T4 trajectories, showing that these phages adhere to mucin glycoproteins and exhibit subdiffusive motion within mucus^101^. This increases the frequency of bacterial encounters with phages as cells transit through mucus, thus potentially aiding the immune system in clearing bacterial pathogens^98^. Their later work showed that eukaryotic cells can internalize phage T4 particles and use them as a carbon source^100,107^. More recently, Zamora and colleagues examined therapeutic phages that target *Pseudomonas aeruginosa*, and tested how these particles enter human-derived airway epithelial cells^108^. The virus particles were labeled with a fluorescent dye, and their particle size distribution was measured through analysis of 3D images acquired through confocal Z-stacks. They found that phage particles translocate to areas of epithelial remodeling without disrupting cellular integrity, and that this translocation as well as degradation was dependent on the phage species^108^. We expect heterogeneity in this interaction for phage genotypes within a population. While the studies discussed here did not focus on the intraspecies variation in the interaction, they used approaches visualizing individual viral particles and thus paved the way to study the intraspecies variation. Understanding how and why phage particles enter human cells, and their variation in doing so, seems important for concerns over reduced ‘delivery’ (dosing) during phage therapy as well as off target biological effects that can alter human or other animal host immunobiology such as interferon production, anti-phage antibody production, and other immune-defense mechanism priming.

## Conclusions and open questions

Most traditional approaches to measure phage-bacteria interactions rely on the classic double-agar-layer plaque assay. This method can produce false positives (e.g., lysis from without, where concentrated phage particles disrupt bacterial growth without completing their replication cycles) and, more commonly, false negatives (failure to form visible plaques despite successful phage replication) in non-model systems where double-layer plaques may not reliably indicate phage growth^109,110^. These assays are optimized for lytic phages (the focus of this perspective), which represent additional challenges when working with temperate or filamentous phages. Crucially, these classic approaches provide estimates of the average traits for individuals in these microbial populations. Here we argue that variation in virus traits among particles from the same population should be studied more intensively, as variation within populations and species is important for elucidating critical phenomena in biology^21^. Broadly, the fields of optical microscopy and flow cytometry offer particularly compelling platforms to develop new or better approaches for measuring individual-level variation in phage-bacteria interactions. Rather than providing a thorough review of such approaches, our goal was to pique interest in the topic, and below we suggest many open questions and perhaps fruitful areas of future research efforts.

To motivate further studies, we include a non-exhaustive list of some open questions relevant for phage biology and biotechnology development.

### Open questions in phage biology

- What new insights into phage biology could emerge from accurately measuring traits of individual particles rather than population averages?
- How much trait variation exists among individuals in a phage population and what evolutionary forces shape this variation?
- In the proverbial gram of soil or drop of seawater, how much does individual particle performance vary within the same population?
- Is phenotypic variation relatively narrow (canalized) for some fitness components (e.g., certain stages of the phage replication cycle) but broad for others?
- Can accurate measurement of phenotypic variance and covariance for phage traits across environments help us better partition total phenotypic variation into its environmental and genetic components?
- Do phages evolve to exploit cell-surface receptors based on their numerical abundance, spatial configuration, and/or structural invariance on host bacterial cells? What role does phenotypic variation in host bacteria play in this evolution?
- How does particle stability (often termed as half-life) affect infection ability across all replication stages, from entry through progeny release?
- How do these relationships change when the host cell’s metabolism operates in warmer, drier or other atypical/stressful conditions, including those inferred for Earth’s past geological history or predicted to change in the Anthropocene?

### Open questions to aid phage-based biotechnology

Much like other domesticated biological systems, leveraging phage traits to solve human problems depends on available phenotypic variation and our ability to distinguish useful variants. Different traits may be desirable for different end-goals in technological development. For example, phages with short half-lives might be better for decontaminating a water reservoir, thereby reducing phage ability to subsequently spread (emerge) on other bacteria in the environment. Whereas, longer lived phages might be preferred as genetic vectors for engineering bacteria in situ, such as recombining into host bacteria that naturally associate with cancer tumors, causing these prokaryotic cells to locally secrete anti-tumor toxins. Human health relies on maintaining the cleanliness of built environments, whether rural hospitals or enclosures on the Moon and Mars, and phages may be useful as decontaminants. Again, phenotypic variation within phage populations may significantly affect these intended outcomes^75,76^, and hence warrants careful scrutiny.

- Could next generation decontaminants contain phages engineered to withstand chemical disinfectants while targeting bacteria that survive standard chemical treatments?
- Can phages be engineered to attack multiple antibiotic-resistant bacterial species by exploiting conjugative pili from multi-drug-resistance plasmids?
- How can we maintain therapeutic efficacy while minimizing off-target effects in clinical applications?

These applications would benefit from basic research that elucidates how and why phage variants differ in fitness-related traits, including particle stability, binding kinetics, replication efficiency, and ability to maintain these and other interacting traits when used as vectors in recombinant technology. The high-throughput approaches reviewed here can guide biotechnology development, such as identifying optimal phage candidates for bacterial diagnostics, bacterial tracking via prophage insertion, and therapeutic application in humans, animals, plants, etc. Moreover, product consistency remains challenging in phage biotechnology due to inevitable genetic variation from spontaneous mutations. A key goal is to harness this variation constructively—minimizing detrimental phenotypic effects while preserving beneficial traits. Studies examining fitness variation among individual particles, coupled with improved measurement tools, can inform strategies for managing variation and accelerating progress toward ambitious goals in biotechnology.

## Supplementary information

Supplementary information

## Data Availability

No datasets were generated or analysed during the current study.
