# Supplementary material for: Rage against the mean: a perspective on measuring fitness of individual phage particles
Source: Npj Viruses. 2026 Apr 4;4:21. doi: 10.1038/s44298-026-00187-4 (PMC13050327; doi:10.1038/s44298-026-00187-4)

Rage Against the Mean: A Perspective on Measuring Fitness of Individual Phage Particles

Jyot D. Antani[1,2,3,✉], Paul E. Turner[1,2,3,4,✉]

[1]Department of Ecology and Evolutionary Biology, Yale University, New Haven, CT 06520, USA

[2]Center for Phage Biology & Therapy, Yale University, New Haven, CT 06520, USA

[3]Quantitative Biology Institute, Yale University, New Haven, CT 06520, USA

[4]Program in Microbiology, Yale School of Medicine, New Haven, CT 06520, USA

✉ Corresponding authors: jyot.antani@yale.edu; paul.turner@yale.edu

Supplementary Information

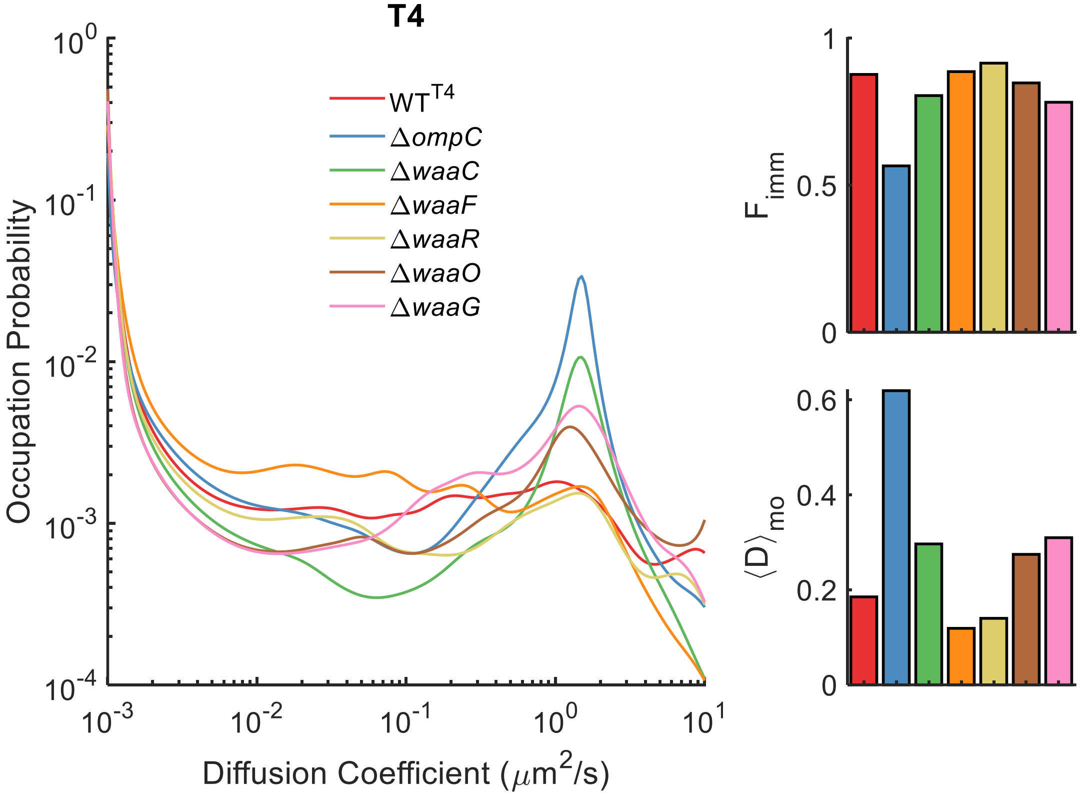

Fig S1. Machine-learning-based inference of biophysical parameters of phage binding (part 1/2). saSPT analysis (Heckert et al., *eLife*, 2022) was performed on the phage trajectories published in our earlier work (Antani et al., *PNAS*, 2024), to study target search mechanisms by phages. For each phage, three panels indicate *[Left]* distributions of inferred diffusion coefficients *[Right, top]* fraction of immobile (bound)

phage trajectories defined as $P(D < 0.05\ \mu m^2/s)$ *[Right, bottom]* Mean free diffusion coefficient based on the above threshold. See **Fig S1 (part 2/2)** below.

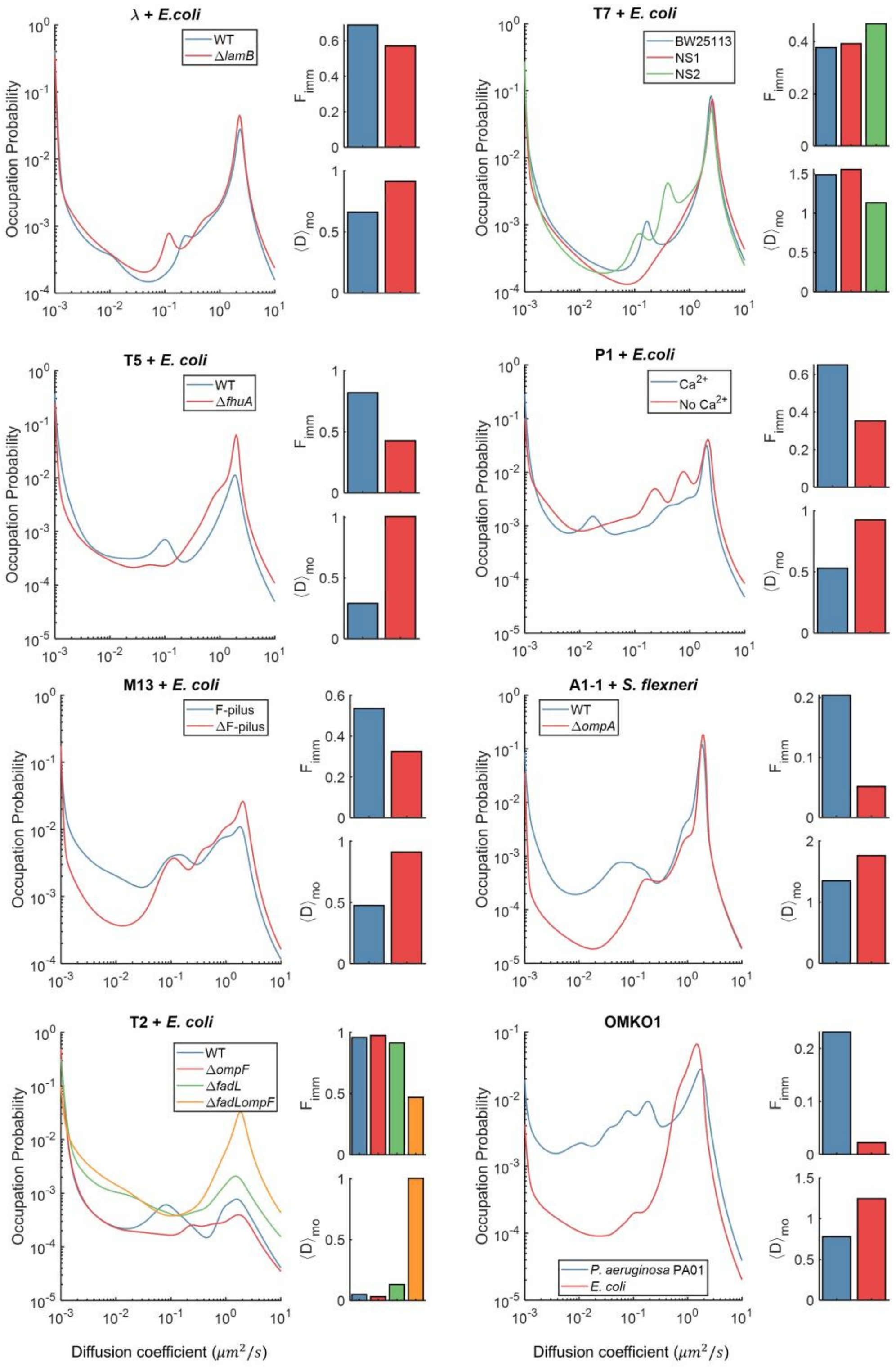

Supplement: Supplementary file 1 — Supplementary information [file 44298_2026_187_MOESM1_ESM.pdf]
